# Addiction and autonomy: Why emotional dysregulation in addiction impairs autonomy and why it matters

**DOI:** 10.3389/fpsyg.2023.1081810

**Published:** 2023-02-06

**Authors:** Edmund Henden

**Affiliations:** Centre for the Study of Professions, Oslo Metropolitan University, Oslo, Norway

**Keywords:** addiction, autonomy, value, self-control, emotional dysregulation, motivation, decision-making capacity

## Abstract

An important philosophical issue in the study of addiction is what difference the fact that a person is addicted makes to attributions of autonomy (and responsibility) to their drug-oriented behavior. In spite of accumulating evidence suggesting the role of emotional dysregulation in understanding addiction, it has received surprisingly little attention in the debate about this issue. I claim that, as a result, an important aspect of the autonomy impairment of many addicted individuals has been largely overlooked. A widely shared assumption in the philosophical literature is that for addiction to impair a person’s autonomy it has to make them (in some sense) take drugs against their will. So-called “willing addicts” are therefore usually seen as exempted from the autonomy impairment believed to characterize “unwilling addicts,” the latter being those who “truly want” to stop using drugs but find their attempts repeatedly derailed by failures of self-control. In this article, I argue that the association between addiction and emotional dysregulation shows why this assumption is false. Emotional dysregulation is not only consistent with the possibility that many addicts take drugs “willingly,” it supports the hypothesis that they use drugs because they truly want to. The article proposes an explanation for why emotional dysregulation should nevertheless be seen as an aspect of their loss of control and an important reason why they have impaired autonomy. I end by exploring some implications of this account for addict’s decision-making capacity when they are prescribed the drugs to which they are addicted.

## Addiction, philosophy, and emotional dysregulation

In recent decades addiction has become an important topic in philosophy. In severe cases addictions typically lead to actions that create a wide range of problems in the life of the addicted person as well as in the lives of people affected by their actions. Actions that harm not only the person who performs them but also other people, tend to trigger moral responses in us. Either we criticize and attribute blame, or we sympathize and seek to help. Which one it is depends to a large extent on what we consider the likely causes of the actions. If we believe the cause is the person’s own *will*, we are inclined to criticize or blame him. If instead we believe the cause is external to the person’s will – something over which he had little or no control – we are inclined to sympathize with him and endeavor to help. For example, if a person lashes out verbally and grabs your arm, the fact that they suffer from a brain disease like Alzheimer makes a difference to what moral response is required. If you are like most people you think it inappropriate to blame him because you realize that the cause of his aggressive behavior was not really *him*, but his disease, which he cannot control.

Suppose, then, that a person is addicted to a drug and that this fact leads him to do things that not only harm himself but also other people around him. What is the moral weight of the fact that he is *addicted*? Should this fact make any difference to attributions of blame or fault, and if so, what is it about this fact that produces this difference? While the case, of course, is less clear cut than the Alzheimer’s example, it is still reasonable to ask to what degree the person’s will was the cause of his actions, and to what degree the cause was his addiction. According to a long philosophical tradition, actions caused by the agent’s will are those she performs autonomously and should be held morally responsible for Kant (1997). Much of the contemporary philosophical debate about addiction has therefore revolved around questions of the extent to which actions stemming from addiction should count as autonomous, or how far the addicted person should be held morally responsible for them (Watson, 1999; Charland, 2002; Yaffe, 2002; Foddy and Savulescu, 2006; Levy, 2006; Morse, 2011; Schroeder and Arpaly, 2013; Pickard, 2017; Matthews and Kennett, 2019). Unsurprisingly, the controversy surrounding these philosophical questions has been fueled by disagreement over terms such as “autonomy” and “moral responsibility,” and even more so by a lack of general philosophical and scientific consensus about the nature and definition of addiction. In an influential paper on autonomy and addiction, Levy, for example, after having rejected the view that addiction is a brain disease, claims: “Once we recognize that addiction is not incompatible with choice or volition, it becomes clear that none of the standard accounts of autonomy can satisfactorily explain the way in which it undermines fully autonomous agency” (Levy, 2006, p. 427). Despite thinking that addicts “take drugs because they want to,” Levy suggests there still is a sense in which it is true (as they themselves frequently claim) that they “consume against their will,” and that they therefore have impaired autonomy (Levy, 2006, p. 433). Other philosophers, however, like Foddy and Savulescu, find little reason to think addiction impairs addicts’ autonomy (Foddy and Savulescu, 2006, 2010). Given the incredible stigma which drug use has, it is reasonable, they claim, to be skeptical about addicts’ assertion that they consume against their will. In their view, addictions are nothing other than normal but strong desires for pleasure, and such desires do not significantly reduce autonomy even if they can be harmful to the individual.

The debate over addicts’ autonomy is not only of concern to philosophers interested in abstract notions of autonomy and moral responsibility. It has real-world repercussions. If, contrary to popular belief, there are in fact no good reasons to think that addicts’ choices to use drugs are any less autonomous than the choices of most non-addicts who choose to pursue pleasurable goods and activities, this should have dramatic consequences for a wide range of issues, ranging from drug treatment and intervention policies to legal assignment of responsibility. In spite of accumulating evidence that emotional dysregulation is a common driver behind addictive behavior, it has received surprisingly little attention in this debate. One claim I will make is that, as a consequence, an important aspect of the autonomy impairment that is characteristic of many addicts has been largely overlooked. A widely accepted assumption in the autonomy literature has been that for addiction to impair a person’s autonomy it has to *make* them (in some sense) consume against their will. So-called “willing addicts” are therefore usually seen as exempted from the autonomy impairment characterizing “unwilling addicts.” While the former term refers to addicts who use drugs because they “truly want” to, the latter refers to those who “truly want” to stop but take them in spite of this because they fail to resist or refrain from acting on powerful drug desires. Hence, the reason some philosophers, like Foddy and Savulescu, reject the view that addiction impairs autonomy is because they believe that few addicts, if any, use drugs despite truly wanting to stop. In their view, most individuals addicted to drugs use them because they really want to. They are, in this sense, “willing addicts.” Their use of drugs should therefore be treated as a fully autonomous choice.

I believe Foddy and Savulescu are right that many addicted individuals probably are willing addicts. However, the conclusion they draw from this is mistaken. Even if many addicts consume drugs “willingly,” it does not necessarily follow that their drug use is a fully autonomous choice. The source of the mistake is the assumption that addiction impairs autonomy only if it makes addicts consume *against* their will. In this article, I argue that the association between addiction and emotional dysregulation shows why this assumption is false. Emotional dysregulation does not necessarily remove the addict’s capacity to choose or cause them to consume against their will. Nevertheless, I shall argue, it is a crucial aspect of many addicts’ loss of control and part of what explains why they have impaired autonomy. The claim put forth here is that emotional dysregulation undermines important preconditions for autonomous preference-formation, and that, over time, this can lead to a reorientation in values which makes the values associated with *the emotion-related role* drug use has in the addict’s life, central to their conception of the good. The article ends by exploring some implications of this view for the ethical debate about addicts’ capacity to consent to take part in clinical research which involves giving them their drug of choice.

Before starting, some clarifications are in order. First, in line with standard usage in the philosophical literature, I use the terms “addict” and “addiction” throughout. It should be noted that these terms are absent from the official DSM-5 substance-use disorder diagnostic terminology (although the reasons for omitting them are a matter of some controversy, see Charland, 2020). I will not attempt to provide a very precise definition of “addiction” here. Following other writers, I use this term to describe drug-seeking and use that is characterized by psychological feelings of craving and impaired control and where use persists despite negative consequences (Husak, 2013). Second, addictive motivation operates within a complex causal structure involving a diverse number of interacting influences, ranging from different social conditions, through the types of drugs used, to personal resources and other internal factors, all of which may diminish (or enhance) addicts’ autonomy in different ways. The extent to which addiction impairs addicts’ autonomy is therefore likely to vary, both between individual addicts and across different groups of addicts. I am not claiming that the autonomy of all addicts is significantly compromised. Addictions vary in terms of severity, and it is plausible that those only mildly addicted retain substantive autonomy over their drug use. The focus of this paper is on that subset of addicts labeled in the clinical literature as having “severe” addiction, i.e., individuals who exhibit six or more of the symptoms listed in DSM-5. It also bears emphasizing that I am not assuming that emotional dysregulation is the *only* factor that might explain the manner in which addiction impairs autonomy. No doubt many different factors and mechanisms might contribute to explaining this impairment (in combination with emotional dysregulation). My concern is the normative significance of the claim that emotional dysregulation is part of what lies behind the drug-oriented behavior of many addicts. Exactly what the relation is between emotional dysregulation and addiction (whether it is a cause, correlate, or consequence) I take to be a matter for empirical addiction science to determine.

## Autonomy, addiction, and temptation

Autonomy (*autos* – self; *nomos* – law) refers, very generally, to the capacity to govern oneself free from external or internal interference. When determining the autonomy of an action, it is useful to think of autonomy in terms of the agent’s governing of the process through which she makes the decision to perform that action (Buss, 2012). A decision-making process, in the most basic sense, involves forming an intention (or goal) and selecting an action to satisfy that intention (or achieve that goal). For this process to be properly governed the agent must therefore have some degree of control over the forming of her intention (or goal) and over whether the selected action will satisfy that intention (or achieve the goal). I will be assuming in this article that the relevant form of control can be characterized in terms of possession of a set of *competencies* that legitimizes respecting the agent’s decision to perform the action, and which places valid limits on what others might do to him (Beauchamp, 2005). This set includes the competence to comprehend the action, the competence to reflect on whether or not to perform the action, and the competence to effectively implement the decision to perform the action.

In the context of this competency view of autonomy, “comprehension” is generally understood to refer to the competence to recognize basic facts about one’s action, such as what kind of action it is and what is likely to follow from performing or not performing it (Berofsky, 1995; Mele, 1995; Beauchamp, 2005; Killmister, 2013). Influences that interfere with comprehension in this sense might undermine autonomy by reducing control over the forming of one’s own intentions or the setting of one’s own goals (as in cases of manipulation or brainwashing), or by reducing control of the effects of one’s action on the achievement of one’s goals (as in cases involving false beliefs about what one is doing). “Reflection” is usually understood as involving the competence to step back and rationally assess motives, and to determine which one provides the most compelling reason to act. Without the ability to reflect one would be at the mercy of whichever proximal desire or urge happens to have the upper hand at any given time (Frankfurt, 1971; Dworkin, 1988). In line with a common view in the autonomy literature, I will assume that to be able to govern oneself with respect to such desires or urges, one needs to form – through reflection – a normative conception of oneself in relation to the world. This normative conception expresses one’s reflective (or “genuine”) self and consists of the set of considerations, principles, or values one has of what constitutes a good life (for different versions of this view, see, e.g., Watson, 1975; Ekstrom, 1993; Smith, 2005; Bratman, 2007). Hence, influences that interfere with reflection might undermine autonomy by preventing a person from tracking these considerations, principles, or values in their practical reasoning. The result is reduced control over the contents of one’s practical judgements about what to do (i.e., what one judges “best” or evaluates as the most important. For example, under the sway of a strong feeling one might greatly exaggerate the value of doing something that one later regrets). Finally, the notion of “effective implementation” refers to the competence to execute the decisions one makes based on one’s practical judgments (Naik et al., 2009; Killmister, 2013; Levy, 2016; Matthews and Kennett, 2019). Influences that interfere with this competency might undermine autonomy by reducing control over what one does relative to what one *decides* to do (e.g., one fails to follow through on one’s decisions to do what one judges best or evaluates as the wisest course of action).

Assuming the competency view, how does addiction impact on addicts’ autonomy? Much of the philosophical debate around this question has been shaped by the assumption that drugs constitute a very powerful *temptation* for addicts, a temptation to which most of them usually succumb. Supporting this is the common observation that many addicts report they want to stop using drugs, that they struggle mightily against the temptation to consume, and that they typically fail to resist this temptation (especially in the presence of drugs or cues predicting the drug’s availability). For an individual to be tempted, the object of temptation must have some positive valence that makes him want it very much. Addictive drugs clearly satisfy this condition. However, temptation implies more than just wanting. It is also normative notion. Often we do things we want without experiencing anything that in ordinary parlance could be described as a “temptation.” For example, when I grab a glass of water, I (usually) do so not because I am succumbing to a temptation but because I am thirsty and simply want water very much. A common view is therefore that temptation implies, in addition to wanting, that one believes that what one wants is somehow wrong (Day, 1993). As Orlandi and Stroud put this point, temptation always involves some form of ambivalence on the part of the tempted individual: it implies that her actions, choices or decisions are “influenced by a factor whose influence she rejects” (Orlandi and Stroud, 2021, p. 228). This is why temptation can be said to be the occasion for the exercise of what may be called *reflective self-control*. The view that addicts succumb to the temptation to consume therefore suggests that they frequently *fail* to exercise reflective self-control.

As I understand it, reflective self-control is constituted by intentional efforts to bring one’s actions into line with what one truly wants to do when faced with competing (either occurrent or anticipated) desires or urges. The content of the notion “truly wants to do” has been understood differently by philosophers, but most prominently perhaps, it is seen as expressing a second-order volition, an “all things considered” better judgment, or the long-standing preferences or values that unify one’s agency over time (Frankfurt, 1971; Watson, 1975; Kennett and Matthews, 2003; Levy, 2006; Bratman, 2007). Addicts, it is assumed, typically fail to exercise reflective self-control in one or another of these senses, i.e., when faced with a drug use opportunity, they fail to stick either to their second-order volition, their better judgement, or long-standing preferences or values. Ambivalence is therefore often thought to be the hallmark of addiction: addicts typically take drugs while at the same time *rejecting*, from *some* reflective point of view, the influence their drug desires exert on their actions, choices or decisions. In terms of the competency view, this motivational influence therefore undermines autonomy by disrupting or interfering with their competence to act in line with their normative and/or reflective selves. The resulting theories of addicts’ autonomy impairment can be called *mismatch* theories.

Very generally, there are two types of mismatch theories. First, there are “synchronic” mismatch theories. In these theories the addict’s failures are conceptualized in terms of a synchronic mismatch between what the addict (in some sense) truly wants to do at the time of choice (which is to stay abstinent) and what he chooses to do at that time (which is to take drugs). A classic example is Frankfurt (1971) who assumed that the addict at the time of choice typically has a second-order desire to be moved by a first-order desire to abstain from drugs, but his first-order desire for drugs moves him to take them regardless of his second-order desire. Frankfurt hypothesized that the reason for this is that the addict’s first-order desire for drugs is an irresistible temptation that overwhelms his capacity to choose. Of course, not all cases of addiction are characterized by mismatches of the above kind. Addicts who do not experience any ambivalence are what Frankfurt called “willing addicts.” The willing addict, he writes, is someone who is “altogether delighted with his condition … who would not have things any other way. If the grip of his addiction should somehow weaken, he would do whatever he could to reinstate it” (Frankfurt, 1971, p. 19). As Frankfurt understood this, it meant that he has aligned both his first and second-order desires with each other so that he fully endorses his drug-oriented choice after critical reflection. It has been common in the philosophical autonomy literature to view the addict who, in this sense, “want what he wants to want” (p. 15) – *the willing addict* – as a fully autonomous agent.

Second, there are “diachronic” mismatch theories. In these theories the addict’s failures are conceptualized in terms of a diachronic mismatch between what the addict (in some sense) truly wants to do (which is to stay abstinent) and what he chooses to do at the time of choice (which is to take drugs). An important part of the reason why the most influential contemporary theories belong in this category is a growing consensus in the literature that it’s empirically false that drug desires are irresistible temptations that efface the addict’s capacity to choose (Foddy and Savulescu, 2006; Levy, 2006; Pickard, 2015; Sripada, 2018). This is supported by evidence that addicts are responsive to various incentives to abstain from drugs, that many give up drugs voluntarily (for a variety of reasons), and that drug use typically involves complex behaviors that bear all the signs of being the outcome of choice processes: a deliberate weighing up of the costs and benefits of different options, rather than drug desires operating independently of such processes. Usually, the point of departure for diachronic mismatch theories is the dynamic choice literature, where temptation is understood as a temporary shift in an agent’s preferences. As Ainslie (2001) and other choice theorists have argued, addicts experience frequent preference reversals because they tend to discount the utility of future rewards hyperbolically. Levy (2006), combining Ainslie’s theory with Holton’s (2004) account of how temptations may cause “judgment-shifts” which undermine “strength of will” (a special kind of resolution in Holton’s view), hypothesizes that hyperbolic discounting is likely to make addicts vulnerable to regular and uncontrollable judgment-shifts: when the opportunity for consumption is some time in the future, they judge abstinence to be, all things considered, better than use, but as the opportunity draws closer, they unreasonably shift to the judgment that use is better than abstinence. Such judgment-shifts prevent them from “extending their will across time,” i.e., putting their long-standing preferences (those which unify their agency and reflect what they truly value) into effect. According to Levy, even if addicts choose to consume because they want to, addiction therefore still impairs their autonomy because it prevents them from sufficiently integrating their agency over time in order to do what they truly want to do (which is to abstain from drugs). In a recent article, Matthews and Kennett also connect addicts’ autonomy impairment to a failure to unify their agency over time, noting that, “What strikes us is that … the addicted agent cannot effectively execute their decisions to stop using. These failures mean they cannot live according to their values” (Matthews and Kennett, 2019, p. 53). Matthews and Kennett suggest the reason for this is resignation and lack of self-trust after years of failed attempts to get their lives back on track. Thus, many addicts, they claim, just give up: “They share common views about the constituents of a good life, but they estimate that such a life is not open to *them*” (p. 54).

For me, the problem with these mismatch theories is not that they (necessarily) fail to explain the autonomy impairment of addicts who truly want to stop using drugs, but that they fail to explain the autonomy impairment of those who *do not* truly want to stop using them. The former category includes addicts who, in Frankfurt’s words, “hate [their] addiction and always struggle desperately … against its thrust” (Frankfurt, 1971, p. 12). They are “unwilling” because they consume drugs despite wanting to quit and trying hard to exercise self-control. The difficulty they face is being successful *in resisting or refraining* from acting on their desires for drugs. However, although many addicted individuals are without doubt “unwilling addicts” in this sense, many are not. These individuals make little effort to exercise restraint, nor do they seek help for their addictions. A report from the U.S. Department of Health and Human Services estimated that in 2006 only 4.5% of the 21.1 million people classified as needing, but not receiving, substance use treatment reported a perceived need for therapy (Goldstein et al., 2009). Evidence like this makes it plausible to speculate that the difficulty facing many addicts may be more one of motivating themselves to try to exercise self-control – to make up their mind to quit drugs and change their lives accordingly – than successfully exercising such self-control assuming they already have this motivation. Is this always because of resignation and lack of self-trust? If they “hate” their addiction that, of course, could be a plausible explanation (otherwise, why do they not just stop?), but here I want to explore another possibility. In a recent article, Pickard argues that addiction cannot be explained without recognizing *the tremendous value* drugs have to those addicted (Pickard, 2021). I believe this is right. In fact, the value drugs have for many addicts could be part of the reason why they make little effort to exercise restraint and seek help or treatment. In my view, the major shortcoming of mismatch theories is that they overlook the significance of this value in explaining the loss of autonomy that characterizes severely addicted individuals.

## The role and value of addictive drugs

There are many familiar reasons to think that addicts place a high value on drug use. As Foddy and Savulescu (2006) point out, addictive drugs are similar to food and sex in that they are reliable sources of pleasure. Although it is debatable how much pleasure addicts get from consumption (especially after longer-term use), it would seem implausible to rule out that pleasure is one common and important constituent of the good they get from drugs. But the value drugs have for addicts extends far beyond pleasure, narrowly construed. It is well-known that addicts consume drugs for a variety of reasons: to drown out anxiety, minimize stress, relieve pain, enhance self-confidence, and many others (Müller and Schumann, 2011; Pickard, 2012). As Lewis (2011) writes, “One way or another, whether they are junkies or executives, people take drugs because they are not feeling right. The whole point of taking drugs is to change the way you feel” (Lewis, 2011, p. 38). In clinical approaches to addiction, it is widely assumed that an important function of drug use is to regulate emotions, to alleviate negative emotions and increase positive ones (Gratz and Roemer, 2004; Berking et al., 2011; Kelly and Bardo, 2016; Rodriguez et al., 2019). I will not attempt to provide a definition of emotion here, but shall use this term in a relatively broad sense, in line with the way it is commonly used in the emotion-regulation literature, as referring to a wide range of psychological states or processes from more simple ones like “joy,” “sadness,” “anger,” or “fear” to more complex ones like “shame,” “guilt,” “regret” and so on. Now, given the plausible assumption that people, in general, regard it as valuable to be in certain emotional states (e.g., feeling confident, content, calm etc.), and not valuable to be in other emotional states (e.g., feeling anxious, sad, restless etc.), the fulfillment of the emotional regulatory function is plausibly also an important part of the value addicts get from drugs. Yet, there has been surprisingly little exploration in the philosophical literature of what this might mean for the question of addicts’ *autonomy*. One exception is Matthews and Kennett (2019) who recognize that drugs typically have this function and acknowledge that it can be a source of value for many addicts. However, they suppose that the values it creates are “synchronic” (temporary or current) and contrast them with the “diachronic” (long-standing or enduring) values that constitute the addict’s conception of the good. The resigned addict, they argue, remains alive to the diachronic values their addiction denies them, but the believed unavailability of these values lead them to focus on available synchronic values instead, such as “temporary relief, or escape, or the simulacrum of social connectedness” (p. 55).

This argument seems to assume that there is a sharp line between an addict’s drug-oriented values and the diachronic values that comprise their reflective selves (those that are internal to their conception of the good). However, it is not clear why the regulatory function drug use has for many addicts cannot also become a source of diachronic values for them. In support of this it might be pointed out that it is their *use* of drugs that performs this function. Of course, addictive drug use includes consumption of the drugs and the concomitant mental and bodily effects (which are temporary or current). But it is not limited to these effects. It is a recurrent pattern of behavior that takes place over time and in contexts which involve objects, practices, rituals, and a community of other users, all of which might contribute (by association) to the fulfillment of this regulatory function, i.e., become a means to alleviate or increase negative or positive emotions. Consequently, all these things can be imbued with positive emotional meaning and so become valuable to the addict. Clearly, the resulting values are not all about the temporary rewards of consumption but might be connected to a diverse set of rewards obtained from *the emotion-related role* drug use has in their life. This role can be associated with many different goals (in addition to those already mentioned), including providing a sense of purpose and structure in daily life (many addicts spend most of their waking hours either thinking about, planning or seeking the next fix), a sense of belonging and acceptance within a drug community (for many addicts the pathway into addiction involves a lack of social relationships and support), and sometimes even a sense of self and identity as an addict (Dingle et al., 2015; Flanagan, 2019; Pickard, 2021). As Flanagan writes, addiction can become “a lifestyle … that involves deep identification with aspects of the very kind of life that is … out-of-control” (Flanagan, 2019, p. 87). In light of this it would seem implausible to rule out that values associated with the emotion-related role drug use plays can become part of what embeds a cross-temporal structure on addicts’ thoughts and behavior (especially in long-term addictions).

But this raises a question about which values are more central to the addict’s self. Diachronic mismatch accounts assume these are the values they had *before* they became addicted, that these values remain unchanged throughout their addiction, and that their loss of autonomy should be defined in terms of their failure to stick to these values. But there does not seem to be any way of determining whether these values are in fact more central. One line of thought could be as follows: the values associated with the emotion-related role drug use has for many addicts probably dispose them to believe that drugs are something they *need*, not just to satisfy temporary drug desires but, more generally, to cope with everyday life. If such a belief is part of how they experience their addiction, there is little reason to expect that they will *reject* the influence exerted by their drug desires on their actions or decisions, even if they were to reflect upon it from the perspective of values they had before they became addicted. To the contrary, a reasonable assumption could be that the latter values have become usurped by values associated with the emotion-related role drug use plays in their lives. The multiple values associated with this role might, over time, become internal to their conception of the good, such that they have difficulty imagining how to live without them. They might therefore see them as providing reasons for their actions and shape their lives around them accordingly. This line of thought suggests that many addicts use drugs, not in spite of truly wanting to stop, but simply because they *truly want them*. In other words, drugs are not “temptations” for them, rather something they both want very much and genuinely value.

It bears emphasizing that the assumption that many addicts might *not* reject the influence exerted by their drug desires does not imply that they must be like Frankfurt’s willing addict who is “delighted with his condition” (Frankfurt, 1971, p. 19). The fact that a desire is not unwanted (“rejected”) does not mean that one must be delighted with it. To the contrary, it can make one feel quite miserable (think of your desire to go on a painful diet because you believe you “need” to lose weight!). Now, if the addict does not reject the influence exerted by their drug desires from *any* reflective point of view, they plausibly lack a genuine will to stop using drugs. Hence, their drug preferences and judgments in favor of using might be stable across time (at least for extended periods), and they will have no real motivation to exercise self-control to change their addictive lifestyles. They are, in this sense, “willing addicts.” Levy briefly considers the possibility that some addicts are willing in this sense and concludes that their autonomy is *not* impaired by their addiction. He writes, “Addicts lack autonomy when they suffer regular and uncontrollable preference reversals … The addict who always (or usually) prefers consumption …” (and so genuinely *values* a life with drugs) “… is not autonomy-impaired …” (Levy, 2006, p. 440). Foddy and Savulescu (2010) draw a similar conclusion, except they assume most addicts are in fact willing in this sense. As they see it, frequent preference-reversals are common also among non-addicts and only undermine autonomy if one assumes some ideal of autonomous agency that few people live up to in real life. The default assumption should therefore be that most addicts act on desires that are their “considered, most valued priority, and when that priority is a long-standing desire that the person has developed in response to pleasurable experiences, there is no procedural theory of autonomy that should hold the person’s action to be non-autonomous” (Foddy and Savulescu, 2010, p.16).

For me, these claims about the autonomy of “willing addicts” reveal the real limitation of mismatch theories. It is worth observing that there is nothing in the description of these addicts that excludes the possibility that they can be *severely* addicted to drugs, i.e., that they exhibit six or more of the symptoms listed in DSM-5. The “loss of control” widely believed to characterize addicts in this category is not, of course, defined in terms of “failures to live according to their values” or the lack of (some form) of higher-order “endorsement” of their desires for drugs. Whether we judge a person to have control over some pattern of behavior usually depends on an evaluation of how this pattern interacts with the rest of her life (Keane, 2004). This is why we find it difficult, for example, to make sense of the idea that a human agent is in control of behavior that appears outright dysfunctional. The behavioral manifestation of loss of control in addiction is therefore excessive drug intake and the inability to limit drug intake (typically) in combination with subjective feelings of distress and disruption of the ability to function normally in one’s social roles, such as meeting obligations at school, work, and home (Lyvers, 2000; American Psychiatric Associations, 2013). While this may involve a persistent desire or unsuccessful effort to stop using, the absence of such desire or effort is not sufficient to demonstrate the presence of control over drug use (or to rule out that the addict has lost control in the above sense). Consistent with this observation, the *empirical* evidence of addicts’ loss of control comes, as Levy (2006) rightly points out, in large part “from the fact that all too often addicts slowly destroy their lives and the lives of those close to them. They engage in illegal, dangerous or degrading activities in order to procure their drug, they lose their jobs, their partners and their homes” (p. 433). Levy is correct that “[I] f it was *purely* a matter of autonomous choice, we should not expect their lives to spiral out of control so dramatically” (p. 433). But it is noteworthy that the evidence in support of this claim does not by itself imply any particular theory about addicts’ *preferences* or *values*. It does not, for example, exclude that they “always or usually prefer consumption” or “act on desires that are their considered, most valued priority.” Moreover, contrary to what Foddy and Savulescu seem to assume, no *theory* of autonomy is plausibly able to falsify this evidence. Indeed, if some preferred theory of autonomy were to imply that the drug-oriented behavior of these severely addicted individuals (who have clearly lost control over their drug consumption) is purely a matter of autonomous choice, the reasonable inference would be that this would falsify *the theory*, not the claim that their autonomy is impaired as a consequence of addiction. I believe most people would intuitively ascribe diminished autonomy to such individuals.

Given the assumption that the regulatory function of drug use can be a source of value for many addicts, I suggest a plausible view is that, rather than preventing them from sticking to their diachronic values, this function typically prevents them from *abandoning* some of these values. In fact, being in the grip of long-standing values associated with the emotion-related role drug use plays in their lives might be an important aspect of their loss of control. This means that Foddy and Savulescu could be correct when they assert that addicts use drugs because that is their most valued priority. However, it would be a mistake to infer, therefore, that their drug use is purely a matter of fully autonomous choice. In the next section I explain in a little more detail why this inference should not be made.

## Why emotional dysregulation in addiction impairs autonomy

It may be useful to start by distinguishing between reflective self-control and the broader notion of behavioral control. Reflective self-control refers to an agent’s capacity to govern her actions on the basis of some (privileged) evaluative ranking of her options when faced with competing desires or urges. As such, it is subject to formal norms of rationality, i.e., norms which apply to beliefs and preferences, such as the norms of first-order logic, probability theory and expected utility theory (consistency, Bayes rule, transitivity, independence, and so on). The concept of behavioral control differs by being a *functional* notion, in large part defined in terms of psychological processes that have evolved to promote adaptive behavior. Integral to this form of control is a capacity to change and adjust psychological states and responses in relation to one’s contextual situation in order to bring them into line with the demands of the situation and one’s goals in that situation. Since behavioral control, understood in this way, is oriented toward the successful achievement of personal goals in contextual situations, it is arguably subject to a much richer and more diverse set of norms than just the formal norms of rationality. One view might be that this set contains norms which, rather than specifying internal connections between individuals’ beliefs or preferences, identify “correspondences” between people’s psychological states and responses and their social and physical environment (Hammond, 2007). Taking a broad approach to these more substantive norms, they might include norms of empirical accuracy (Gigerenzer, 2000), norms facilitating cooperation and social interaction (McGeer, 2015; Castro, 2020), norms of attention (Fairweather and Montemayor, 2017), norms of well-being (Hsee and Hastie, 2006), norms of human flourishing and proper functioning, the latter is believed by some theoreticians to be central to conceptions of disease and disorder (Wakefield, 1992; Murphy, 2006), and probably many more. For my concerns, the important point is that, assuming this sort of view, the capacity to control behavior in relation to one’s goals and contextual situation must involve a broad set of *skills* or *abilities* for regulating psychological states and responses in accordance with a diverse range of socially shared norms of this kind.

I take emotional regulation (ER) to be an important aspect of behavioral control. In Gross’s influential account, ER refers to the skills or abilities people use to control their emotions. These are exercised through processes aimed at influencing “which emotion we have, when we have them, and how we experience and express these emotions” (Gross, 1998, p. 275). Such processes can be intentionally executed (top-down) and involve mental effort and conscious awareness, but they can also be evoked automatically (bottom-up) and be enacted without mental effort or conscious awareness (Braunstein et al., 2015). Gross distinguishes between five broad categories of ER processes (or strategies) based on where they have their primary impact in the emotion-generating process (Gross, 2014). In the early stages, before the emotion is fully formed, ER processes can increase (or decrease) the probabilities that certain emotions will arise. Examples of such processes include *situation selection* (seeking out or avoiding altogether particular emotion-eliciting situations), *situation modification* (directly altering the situation in order to change the potential emotion it engenders), *attentional deployment* (directing attention to certain aspects of the situation or turning away from aspects that are upsetting), and *cognitive change* (reframing the subjective meaning of a situation in order to change its emotional significance). In the later stages of the process, after the emotion is fully formed, *response modulation* involves directly influencing the emotion in order to change how it is experienced (e.g., taking a deep breath to suppress anger).

As I understand it, while ER is guided by the individual’s desire to change or influence their emotions to increase subjective well-being, *the functional role* of ER is to generate emotional states and responses that match the individual’s social and physical environment. ER is therefore subject to norms of correspondence. When ER works well in accordance with these norms, there will typically be a match between the functional role of ER and the individual’s emotion-related goals, and the emotional states and responses are deemed “adaptive” (i.e., the individual achieves their emotion-related goals, e.g., increased well-being). It is, of course, a difficult matter to give a precise definition of the norms that apply here. As Gross and Jazaieri note, many of them concern the situational appropriateness of different types of emotion, including their expression, duration, intensity, and/or frequency (Gross and Jazaieri, 2014). For example, what would constitute “healthy” grieving depends, in part, on cultural and social norms as to how long, how deep, and how severe the expression of grief should be (Rottenberg and Gross, 2003). When ER works in violation of such norms, there will typically be a mismatch between the functional role of ER and the individual’s emotion-related goals, and the emotional states and responses are judged to be “maladaptive” (i.e., the individual does not achieve their emotion-related goals). The latter is how I understand emotional *dysregulation*.

As the above description from Gross suggests, different types of actions can serve as a means of emotion regulation. Thus, a growing number of studies indicate that drug use serves this function for many addicts (for a review, see Garland et al., 2020). When drug use becomes an individual’s preferred strategy for regulating emotions, it enhances the likelihood that they will seek out situations and people associated with drugs because of the emotions that are generated (e.g., social acceptance, excitement), that their attention will be disproportionally focused on drug-related features of situations because of the emotional impact (e.g., anticipation, pleasure), and that they will rely on drugs to alter negative emotional experience and increase positive emotional experience (decrease emotional discomfort and increase pleasurable feelings). Because of this, many addicts are likely to experience difficulty in disengaging their attention, thoughts, and feelings from drugs. Their practical perspective (what they notice, acknowledge, respond to, pick out as salient, and so on) will therefore be dominated much of the time by drug-related emotions, goals and preferences. One plausible effect could be that *other* emotions, goals and preferences are “crowded out.” As Heyman describes it, using addictive drugs over time may “poison the field, making everything else *relatively worse*” (Heyman, 2009, p. 145. My italics). Evidence of this is that addicts typically experience a loss of interest in things and activities that they used to value, such as hobbies or spending time with family or friends. But what explains this change in valuing? Consider the common philosophical view that emotions disclose or overlap with values. It is beyond the scope of this article to expound the many different theories about the nature of this connection, but one shared idea is that emotions present the world evaluatively to us, either because they are a kind of judgment of valuable states of affaires, or a kind of perception of such states (Solomon, 1976; Lyons, 1980; Nussbaum, 2002; Prinz, 2004; Tappolet, 2016). Now, it seems reasonable to assume that by dramatically *increasing* the emotional salience of everything related to drugs prolonged drug use correspondingly *decreases* the emotional salience of everything *not* related to drugs. In conjunction with the view that emotions present the world evaluatively to us, one plausible hypothesis could be that this mechanism causes, over time, an *erosion* of values associated with goods and activities that compete with drug use.

The “crowding out” effect might explain why Foddy and Savulescu could be correct that many addicts use drugs because it is their most valued priority. But rather than being the result of regular volitional processes, it would be the result of *a loss of control* over the emotion-generating process. Thus, when drug use acquires a regulatory function, in particular when it becomes a means to suppress negative emotions, it can lead to a downward spiral toward compulsive usage patterns. The addict systematically grasps for this strategy to stave off negative emotions, but as the effects of the drugs wane, the negative emotions return, more of them rather than less, and often worse than before. The addict’s response is to use more drugs, thereby causing them to experience even more negative emotions. The consequence is a vicious cycle that creates more of the very emotions they are trying to avoid, and increases (because of this) the frequency and intensity of their drug cravings, thereby enhancing the dominance of their practical perspective by drug-related emotions, goals and preferences, and so “crowding out” even *more* of their other emotions, goals and preferences. It is easy to see how this process, driven by the addict’s emotional engagement and pattern of reactions to uncontrollable internal events, can culminate in the excessive drug intake and inability to limit drug intake that characterize the loss of control typical of individuals with severe addictions. Because the outcome will be a mismatch between the functional role of ER and the addict’s emotion-related goals, using drugs to regulate emotions is an instance of *dysregulation*. But what difference does such dysregulation make to reflective self-control and autonomy over behavior?

There can be little doubt that emotional *regulation* is an important aspect of both. The reason people often fail to resist or refrain from acting on unwanted desires and urges, is that they are poor at reducing their frequency and intensity through, for example, avoiding particular situations, turning attention to or away from certain features of situations, or reframing the meaning of situations in order to change their emotional significance. Typically, therefore, ER *enhances* reflective self-control, making people more self-controlled (or “continent”) than they would otherwise have been. But even so, ER is not *the same as* reflective self-control. While the occasion for the exercise of reflective self-control is temptation, ER is often exercised in the absence of temptation. People also rely on strategies to shape and guide their emotions when they do not have (or even anticipate having) desires or urges whose influence they reject from any reflective point of view. These strategies might automatically guide their actions most of the time, independently of their own reasons, beliefs, or intentions. ER need not, therefore, involve any intentional effort to bring one’s actions into line with what one truly wants to do in the face of some occurrent or anticipated motivational conflict. In fact, one effect of being good at ER is that there is going to be *less occasion* for the exercise of reflective self-control (i.e., fewer competing desires or urges) and consequently, also less occasion for failures of reflective self-control.

If ER can occur in the absence of temptation, so can emotional dysregulation. That is, people who use maladaptive or faulty strategies to regulate their emotions can do so without necessarily failing to resist or refrain from acting on *unwanted* desires or urges. In other words, the assumption that many addicts might not reject the influence exerted by their drug desires on their actions or decisions (and therefore do not truly want to stop using) is consistent with the possibility that their drug use is driven, in large part, by emotional dysregulation. But if this means that their drug use can be in line with their second-order desires, better judgments, or even long-standing preferences or values – that they, in this sense, use drugs “willingly” – what difference does emotional dysregulation make to their *autonomy*?

First, given the plausible view that reflective self-control is necessary for autonomy and that ER enhances reflective self-control, ER *indirectly* also enhances autonomy. But ER enhances autonomy also in a much more direct way. This can be seen by observing that autonomy requires, not only control over what one does at the time of an action, but also a form of “historical” control over the developmental processes that lead one to perform the action (Christman, 1991; Arneson, 1994; Mele, 1995; Fischer and Ravizza, 1998; Valdman, 2011; Weimer, 2014). Justification for this view typically comes from considering “psychological twins” cases. This is where two people have identical goals, preferences or values at the time of an action, but in one case they are the result of normal character-development processes while in the other they result from some autonomy-undermining influence (e.g., manipulation or brainwashing). In the latter kind of cases, we intuitively think that the person’s goals, preferences or values have arisen in a way that is incompatible with the present autonomy of their action. That is, we think they lack autonomy over their action because they are not autonomous *in relation to their goals*, *preferences or values*.

The philosophical autonomy literature is full of proposals for various abstract conditions that must be satisfied for a person’s goals, preferences and values to be considered “their own.” These range from various constraints on their origins, that the person has carried out some higher-order reflection upon their worth or process of formation, that they have not been acquired in a way that has bypassed the person’s rational capacities, and so on. It is beyond the scope of this article to adjudicate between the different positions in this debate. Instead, I want to suggest that whatever might be (metaphysically) necessary to make goals, preferences and values “one’s own,” there are good *empirical* reasons to think that it depends, to an important degree, on *skills* and *abilities* for regulating psychological states and responses in relation to one’s contextual situations. Put simply, this is because it is through such regulation that people actually appear to manage the developmental processes leading to their actions. That is, evidence from the ER literature suggests that it is by applying strategies such as situation selection, situation modification, attentional deployment, and reappraisal, that people take charge not only of their emotions – but also of their evidence gathering, belief formation and preference development. To give one example: by controlling the movement of attention, one facilitates or inhibits the encoding of information relevant to one’s decision, information that, in turn, might influence not only one’s emotions, but also the frequency, intensity, and longevity of one’s preferences. Moreover, if a person’s genuine values are expressed by a stable set of preferences, this suggests that ER may play an important role in people *shaping* their own values. In sum, according to the view I propose, ER enhances autonomy in a very fundamental way, because it is through the use of ER that people make goals, preferences and values *their own* in appropriate decision-making processes.

Second, if emotional regulation enhances autonomy in this fundamental way, then it is natural to infer that emotional *dysregulation* can diminish autonomy in a similar way. Hence, the emotion-generating process associated with emotional dysregulation is likely to impair the addict’s autonomy with respect to their drug-oriented goals, preferences and values. By “crowding out” everything else, this process prevents significant parts of their motivational system from being sufficiently involved in their decision-making. The claim put forward here is that the consequent lack of involvement of emotions, goals and preferences that are unrelated to drugs might lead, over time, to an erosion of values associated with goods and activities that compete with drug use. This might explain why values and priorities associated with the emotion-related role drug use has in the addict’s life become central to their conception of the good, pushing aside other values and priorities. The effect is likely to impede their ability to vividly and realistically imagine alternatives to drug use and in this way to sap their motivation for change. Importantly, none of this need imply that they have lost the capacity to choose or that they are unable to recognize good reasons to abstain from drugs. Many addicts are undoubtedly aware of the harm their addiction is causing. For an illustration of this point, consider what “John,” an alcoholic in recovery I once interviewed, told me about his situation the first time he came out of rehab, aged 50:

*I had been formally warned by my employer that if I didn’t sober up, I would lose my job. If I lost my job, I would lose my career. I had been told by my doctor that if I continued to drink, my health would suffer severely and death would be a likely outcome. This information was in my head. But it seemed dim, distant, blurry and of little significance. The prospect of life without alcohol was almost unimaginable. It would be at best dull and painful. I had heard from alcoholics in recovery that they were able to enjoy a sober life. But I felt I was different. If alcohol was taken away from me, I would be a sort of husk. So I bought a liter of vodka and returned, with relief, to my life as an alcoholic.*

John was fully aware that he was an alcoholic. He recognized the risks it posed to his career, health and life. Still, these facts had little emotional meaning or salience to him. They seemed “dim, distant and blurry.” What engaged his emotions was the prospect of life *without* alcohol. He worried that such a life would be dull and painful, leaving him with a sense of emptiness inside. John’s response to the negative emotions triggered by the thought of this prospect was to buy a bottle of vodka. There is little reason to think that his decision to return to his life as an alcoholic involved any less information or reflection than many of our decisions do. Still, *the direction* of this decision was heavily influenced by drug-related emotions and values that got in the way of his determining his own action. A few months later, after John had lost his job and ended up in hospital, having (temporarily) lost the use of his legs, he thought he would give abstinence a try. He told me:

*I have not had a drink for eleven years and, to my surprise, I find life without alcohol much better than life with it. I feel at peace and content. Certainly, if you had asked me at 26: “would you choose (a) the life of an alcoholic, drunk every waking hour, incapable of showing up for work, with a high risk of losing your career, physical health and life, over (b) at least a trial period of abstinence” I would have said: “Of course not”.*

The hospitalization and loss of his job may have prompted John’s change of motivation. Prior to this, he might have been what philosophers call a “willing addict”. This article has argued that there is good reason to think that many severely addicted individuals (for shorter or longer periods) are willing addicts like John. Still, their autonomy is impaired as a consequence of addiction. I suggest that this is because their decision-making might be driven by emotional dysregulation which, by undermining the preconditions for autonomous preference-formation, over time, takes charge of their value system. Their loss of autonomy may therefore consist, not so much in a lack of ability to do what they truly want to do, but more in a lack of ability to truly want *a different life*, one that does not involve the regular consumption of addictive drugs.

## Why it matters

The question of whether addicts autonomously choose to use drugs is of great importance to a wide range of issues concerning their proper treatment in society. One example that has received a fair amount of attention in the bioethics literature concerns the capacity of heroin addicts to provide valid informed consent to take part in clinical research on Supervised Injectable Opioid Assisted Treatment (siOAT; Charland, 2002; Foddy and Savulescu, 2006; Henden, 2013; Levy, 2016; Matthews and Kennett, 2019). While the purpose of asking for consent is to protect the right of research subjects to make an autonomous choice, in order to give their consent such potential subjects must be “capable” of making a decision to participate. The question is whether heroin addicts are, in fact, capable of making such a decision, given that participation involves being offered the drugs to which they are addicted (we are assuming they are neither intoxicated nor in withdrawal at the time of consent). Note that having capability here requires more than having a capacity to choose, so possession of the latter does not necessarily imply possession of the former. In medical contexts, four criteria are generally used to assess and determine capability, often referred to as “decision-making capacity”: 1. the ability to understand a choice, such as information about the alternatives, and related risks and benefits; 2. the ability to appreciate a choice, such as grasping the personal relevance of this information; 3. the ability to rationally appraise information, such as evaluating and comparing the risks and benefits of the alternatives in a logical manner; and 4. the ability to communicate a choice (Grisso and Appelbaum, 1998). In addition to these four criteria, many ethicists believe that the decision-maker must, as Buchanan and Brock put it, have “a set of values or conception of the good that is at least minimally consistent, stable, and affirmed as his or her own” (Buchanan and Brock, 1989). This is assumed to follow since in order to evaluate the risks and benefits of alternative outcomes, a certain degree of value stability seems necessary (Charland, 2001; Kluge, 2005; Craigie, 2013).

The criteria for decision-making capacity largely overlap with the competencies held to be necessary for autonomous action by the competency view of autonomy. However, it is important to keep in mind that while the former refers to the capacity to make a particular decision at a particular time and place and is stipulated to be something you either have or do not have, the competencies of autonomy are usually understood more globally as well as in scalar terms. This means that while it is possible that addiction (and emotional dysregulation) diminishes the autonomy of addicted persons to varying degrees, this does not in itself settle the question of whether heroin addicts have decision-making capacity in the context of siOAT’s. Whether they do, depends on how much their autonomy is diminished and what is deemed an appropriate threshold for decision-making capacity in this particular context.

In several influential articles, Louis Charland argued that there are good grounds to doubt the decision-making capacity of addicts in the particular clinical population targeted for heroin prescription trials, namely “individuals with severe treatment-refractory addiction to opiates, who are in dire psychosocial circumstances and suffer from comorbid disorders” (Charland, 2020, p. 8). One of his central arguments concerns the effects of addiction on the capacities that govern emotions. Charland (2002) suggests there is a parallel in how the preferences and values of persons affected by deep depressive feelings may dramatically change, leading them to *underestimate* risks, and how the preferences and values of addicts are affected by feelings tied to pleasure and reward, leading them to *overestimate* the value of drug use. In both cases there is evidence that brain mechanisms underpinning the capacities governing emotions are disrupted. However, while the reorientation in values that occurs in severe depression tends to be of a uniform nature, in the case of addiction it is not. According to Charland, this is due to the dynamics of drug craving, drug seeking, use, and withdrawal, and linked to frequent reversals in preferences. Heroin addicts, he claims, therefore often lack a stable set of preferences and values to guide their decision-making, which suggests they are “unable to manipulate information rationally in the sense required, particularly when decisions about their own heroin use is involved” (Charland, 2002, p. 43).

It is beyond the scope of this article to discuss the brain mechanisms underpinning emotional dysregulation, but there can be little doubt that they disrupt the capacities that govern emotions. Moreover, given the view that there are intimate links between emotions and values, it is plausible to think that such disruptions might affect the addict’s value system. However, in common with many philosophers writing about addiction, Charland’s view of addicts’ autonomy seems predominately shaped by the image of the “unwilling addict” who struggles against unwanted and overwhelming drug desires. Hence, the effects on the addict’s values he seems to have in mind primarily concern effects on their *synchronic* (temporary or current) values. But it is not clear why “fluctuations” in synchronic values must imply that such addicts are “unable to manipulate information rationally.” Such fluctuations seem quite common also among many non-addicts and they are not normally associated with any marked deficiency in rationality. People change their minds about what they ought to do, some so frequently that it may cause them harm, but that does not necessarily render them *irrational* (at least not to any significant degree). Of course, the values that comprise an individual’s reflective self cannot reasonably be assumed to fluctuate in the way synchronic values can. Being stable and enduring is part of their definition. If drug-oriented values can become part of the addict’s conception of the good (as this article has argued), there therefore seems even *less* reason to view addiction *in general* as a deficiency in rationality (which does not rule out that certain forms of ambivalence and irrationality can still be common features of addiction!). From the vantage point of these values, the rational (“best”) thing to do is always going to be to continue to use drugs. However, acting on values that are part of one’s conception of the good is not necessarily sufficient to confer autonomy on one’s actions (as evidenced by cases of manipulation and indoctrination). It also matters how one’s values were formed. The reorientation in values produced by emotional dysregulation is caused by a process that develops independently of the addict’s own direction or guidance and typically leads to *maladaptive* behavior. It can therefore only be understood against the background of the socially shared norms that regulate appropriate decision-making. Hence, it is not the drug-oriented *contents* of these values, but rather their relationship to norm-violating behavioral-developmental processes that explains why they undermine autonomy. But if emotional dysregulation is a normative force that undermines autonomy in this sense, how does it affect addicts’ decision-making capacity?

Several ethicists have argued that emotions are essential to decision-making capacity because they provide us with crucial information about the personal value and meaning of various aspects of our decision-making situations (Cox White, 1994; Silverman, 1997; Charland, 1998). They thus enable us to keep track of our goals and preferences, provide motivation and reasons for our choices, and help us make decisions that reflect our personal values. Given this view, it is reasonable to infer that ER must be important for appreciation of our choices. While “understanding” is said to consist of the ability to receive information, process it, and make it available for use, “appreciation” is understood to consist of the ability to grasp, in a more experiential sense, the personal relevance of this information (Charland, 2001). As Buchanan and Brock describe it, appreciation involves “the ability to appreciate the nature and meaning of potential alternatives – what it would be like and “feel” like to be in possible future states and to undergo various experiences – and to integrate this appreciation into one’s decision-making” (Buchanan and Brock, 1989, p. 24). Examples of conditions discussed in this literature that might undermine appreciation in this sense include excessive levels of fear, anxiety, self-disgust, deep feelings of guilt, hopelessness, worthlessness, and so on (Elliot, 1997; Brown, 2011; Meynen, 2011; Halpern, 2012; Freyenhagen and O’Shea, 2013; Hope et al., 2013). What characterizes such conditions is that they can render us unable to feel differently in the present or to imagine feeling differently in the future (Halpern, 2012). In this way they can prevent us from incorporating essential information into our decision-making.

This article has argued that by “crowding out” everything else, emotional dysregulation prevents anything other than the addict’s drug-oriented emotions, preferences and values from being sufficiently involved in their decision-making, and that this undermines preconditions for autonomous preference-formation. In conjunction with the view that emotions provide us with information about the value and meaning of various aspects of the situations we encounter, this suggests that emotional dysregulation might prevent addicts from incorporating essential information into their decision-making processes. More specifically, the argument supports the hypothesis that emotional dysregulation might impoverish their ability to process information about personally important consequences of alternative courses of action, and thus potentially diminish their decision-making capacity in some circumstances (for evidence of impaired self-awareness in addiction, see Moeller and Goldstein, 2014). In the context of siOATs, this implies that it cannot be excluded that the heroin addicts in the target group might have special difficulties in *appreciating* both what participation as well as *non*-participation in siOATs really means for their own present and future life (e.g., how these alternatives will potentially make a difference to their longer-term goals, future quality of life, and prospects of recovery). If emotional dysregulation is pervasive in severe addiction (as a growing number of studies indicate), Charland therefore seems right to assume that there may be legitimate reasons to doubt the decision-making capacity of addicts in the specific clinical population targeted for siOATs.

## Conclusion

The claim made here is that emotional dysregulation impairs addicts’ autonomy and that it cannot be ruled out that this might negatively impact on their decision-making capacity in situations involving use of their drug of choice. A limitation of the present article is that the justification of this claim largely rests on conceptual and phenomenological arguments that abstract from the circumstances of individual addicts. As pointed out earlier, however, the question of how addiction impairs autonomy cannot be divorced from the question of how it impairs the autonomy of *individual* addicts, and such individuals are bound to differ depending on a multitude of factors, the most pertinent being psychiatric history, social resources, the type of drug used, and so on. Hence, it is to be expected that there will be variations in the autonomy they possess over their drug-oriented behavior. For this reason, we should be careful not to draw any strong conclusion about the autonomy of addicts *in general*, even those who are severely addicted. Charland makes a similar point in relation to the decision-making capacity of heroin addicts earmarked for siOATs. What is needed, he argues, is an evidence-based approach to ethics in this area, noting that “the particular clinical population targeted for siOATs has never been properly clinically investigated for decision-making capacity using up-to-date instruments like the MacCAT-T or MacCAT-CR” (Charland, 2020, p. 8).

Charland’s call for more evidence is reasonable, though it is doubtful (as he himself has argued elsewhere) whether the standardized measures mentioned above take proper account of emotional and valuational factors (Charland, 2006). Ethicists with an emotion-inclusive view of decision-making capacity usually favor a more qualitative and case-based approach, with a stronger focus on the subjects’ personal narratives and experiences (Owen et al., 2009; Charland et al., 2013). Since the normative factors that enter into assessments of the effects of emotional dysregulation on addicts’ decision-making capacity are likely to be nuanced and complex and require substantial interpretation, the latter sort of approach may be more suitable in the context of siOATs. Of course, other factors such as the degree to which the positive benefits to the participants or society outweigh the risks also need to be taken into account in determining the overall ethical appropriateness of siOATs. As Charland (2002) points out, if risks are low and benefits high, one might consider lowering the standards for decision-making capacity or investigate options for surrogate decision-making.

One final comment on a more philosophical note. Some readers might think my criticism of some of the standard accounts of the impact of addiction on autonomy misses the mark because these accounts are procedural, while I assume that autonomy includes substantive and normative elements. In my view, however, this simply betrays the problematic individualism that pervades much contemporary philosophical theorizing about autonomy. As I understand it, human beings are essentially social beings, and for such beings acting autonomously cannot be completely detached from conditions, norms, and practices that foster certain kinds of human relationships. It is beyond the scope of this article to provide a defense of this view, but I hope some of its merits emerge from the way in which it can explain how emotional dysregulation in addiction involves a loss of control that might adversely affect addicts’ autonomy.

## Data availability statement

The original contributions presented in the study are included in the article/supplementary material, further inquiries can be directed to the corresponding author.

## Author contributions

The author confirms being the sole contributor of this work and has approved it for publication.

## Funding

The work presented in this article is part of the project “Autonomy and manipulation: Enhancing consent in the health care context” funded by the Norwegian Research Council (Grant Number: 250503).

## Acknowledgments

For very helpful discussion and comments on earlier drafts of this article, I am grateful to Hannah Altehenger, Sarah Buss, Louis Charland, Jeanette Kennett, and the participants at the workshop “Autonomy in the Healthcare Context”, held at Oslo Metropolitan University. Many thanks also to the editor and reviewers for this journal for their very helpful comments.

## Conflict of interest

The author declares that the research was conducted in the absence of any commercial or financial relationships that could be construed as a potential conflict of interest.

## Publisher’s note

All claims expressed in this article are solely those of the authors and do not necessarily represent those of their affiliated organizations, or those of the publisher, the editors and the reviewers. Any product that may be evaluated in this article, or claim that may be made by its manufacturer, is not guaranteed or endorsed by the publisher.
